# 3066 consecutive Gamma Nails. 12 years experience at a single centre

**DOI:** 10.1186/1471-2474-11-133

**Published:** 2010-06-26

**Authors:** Alicja J Bojan, Claudia Beimel, Andreas Speitling, Gilbert Taglang, Carl Ekholm, Anders Jönsson

**Affiliations:** 1 Department of Orthopaedics, Institute of Clinical Sciences at the Sahlgrenska Academy, University of Gothenburg, Gothenburg, Sweden; 2 Stryker Osteosynthesis, Kiel, Germany; 3 Trauma Unit, University Hospital of Strasbourg, Strasbourg, France

## Abstract

**Background:**

Fixation of trochanteric hip fractures using the Gamma Nail has been performed since 1988 and is today well established and wide-spread. However, a number of reports have raised serious concerns about the implant's complication rate. The main focus has been the increased risk of a subsequent femoral shaft fracture and some authors have argued against its use despite other obvious advantages, when this implant is employed.

Through access to a uniquely large patient data base available, which is available for analysis of trochanteric fractures; we have been able to evaluate the performance of the Gamma Nail over a twelve year period.

**Methods:**

3066 consecutive patients were treated for trochanteric fractures using Gamma Nails between 1990 and 2002 at the Centre de Traumatologie et de l'Orthopedie (CTO), Strasbourg, France. These patients were retrospectively analysed. Information on epidemiological data, intra- and postoperative complications and patients' outcome was retrieved from patient notes. All available radiographs were assessed by a single reviewer (AJB).

**Results:**

The results showed a low complication rate with the use of the Gamma Nail. There were 137 (4.5%) intraoperative fracture-related complications. Moreover 189 (6.2%) complications were detected postoperatively and during follow-up. Cut-out of the lag screw from the femoral head was the most frequent mechanical complication (57 patients, 1.85%), whereas a postoperative femoral shaft fracture occurred in 19 patients (0.6%). Other complications, such as infection, delayed healing/non-union, avascular femoral head necrosis and distal locking problems occurred in 113 patients (3.7%).

**Conclusions:**

The use of the Gamma Nail in trochanteric hip fractures is a safe method with a low complication rate. In particular, a low rate of femoral shaft fractures was reported. The low complication rate reported in this series can probably be explained by strict adherence to a proper surgical technique.

## Background

The Gamma Nail was developed for the treatment of trochanteric hip fractures in the mid 1980's and was first brought into clinical use in 1988. The method of intramedullary nailing evolved from the concepts of Gerhard Küntscher to treat trochanteric fractures [1]. The Gamma Nail development started coincidentally in two places in independent and in parallel processes. It was developed in Halifax, UK, in an attempt to overcome some of the clinical problems with the Zickel nail [2,3] - an intramedullary implant used for the treatment of pathologic subtrochanteric fractures. Simultaneously, a similar implant for same indications was developed at the CTO, Strasbourg, France. These two projects were merged and after a number of clinical evaluations and modifications to both implants and instruments, by 1988 one design emerged designated hereafter as "The Standard Gamma Nail" (SGN).

"The Long Gamma Nail" (LGN) was introduced in 1992 and is used for subtrochanteric hip fractures, femoral shaft fractures and combined trochantero-diaphyseal fractures of the femur. A modified design of the SGN, named "The Trochanteric Gamma Nail" (TGN), was introduced in 1997 and subsequently replaced the SGN.

Today the use of the Gamma Nail is widespread, with more than million patients treated since the introduction of the implant. This is due to several perceived advantages, such as minimal invasive technique allowing for short skin incisions and less blood loss compared with other techniques requiring more surgical exposure [3-6], reduced infection rate, minimal tissue damage, a shorter operating time and early weight bearing [7,8]. The intramedullary position of the Gamma Nail provides a short lever arm for the cephalic screw, still allowing controlled impaction of the fracture [3,4,9-14], but probably with less shortening than with sliding hip screw systems [15].

Despite the widespread use of the Gamma Nail, there are reports on complications that are claimed to be implant-design related [14,16]. The use of the implant has been widely debated because postoperative complications such as subsequent shaft fractures [6,17-19] and lack of scientific evidence supporting intramedullary versus extramedullary technique [3,5,13,20-34]. Nevertheless, this implant has seen numerous competitors [35-41] based on the same concept, i.e. antegrade intramedullary nailing.

We decided to perform a thorough investigation of the Gamma Nail performance over a long period of time focusing on complications. At the Centre de Traumatologie et de l'Orthopedie (CTO) in Strasbourg, France we had access to a large database of thousands of consecutive patients treated with Gamma Nails. In the time period 1990-2002, 3066 consecutive patients treated with Gamma Nails were identified and retrospectively evaluated.

## Methods

The present study is a retrospective analysis of every patient treated with a Gamma Nail at CTO between January 1990 and December 2002. All patients with basocervical (AO/ASIF 31-B2.1), trochanteric (AO/ASIF 31-A), subtrochanteric (AO/ASIF 32-A) or combined trochantero-diaphyseal fractures (Table 1) entering the hospital (CTO) were treated with a Standard Gamma Nail (SGN), a Trochanteric Gamma Nail (TGN) or a Long Gamma Nail (LGN). A small number of patients received another type of Gamma Nails (Gamma-Ti Nail, Long Gamma-Ti Nail, Dyax Asiatic Nail). No other implants were used for these types of fractures during this period. The implants were purchased from Howmedica France S.A. and from 1999 onwards from Stryker France S.A.

**Table 1:** Fracture distribution according to the AO/ASIF fracture classification.

AO/ASIF class	Count	Frequency (%)
31-A1	965	31.5
31-A2	1080	35.2
31-A3	371	12.1
31-B2	170	5.5
32-A	114	3.7
32-B	49	1.6
Other	40	1.4
Missing	277	9.0
Total	3066	100

The patients were treated as surgical emergencies and the procedures were performed both by doctors under training and by senior surgeons. All surgeons were trained for the procedure.

The patients were operated on a traction table in a supine position, both general and spinal anaesthesia were used. Image intensifier was used. Additional fixation such as screws, cerclage wires and bone grafting was used when needed. Full weight bearing was allowed immediately postoperatively, except when the patient was believed to have an insufficiently stable fixation. Radiological examinations were performed pre-operatively, postoperatively within 24 hours after surgery and at follow-up when indicated.

The data collection was performed between 1^st ^of March and 30^th ^of June 2003 at CTO, Strasbourg, France. The study period was from the 1^st ^of January 1990 to the 31^st ^of December 2002. For these 12 years the data were collected for 3066 patients, all treated with Gamma Nails. A separate record of all patients treated with Gamma Nails was kept at the hospital. All available documents of these patients including radiographs were retrieved from the hospital archives. The demographic and technical intraoperative data were completed for all patients with help of the detailed list of all patients treated with Gamma Nail kept at the hospital since the introduction of this implant in the late 1980's.

The medical reports were reviewed for epidemiological data such as age, gender, fracture side, fracture aetiology, co-morbidity, pre- and postoperative mobility levels and presence of pain. Type of anaesthesia, nail type and nail dimensions were recorded. The intra- and postoperative complications were detected with the help of surgical reports, radiographs and follow-up visit notes. Patients were routinely scheduled for a follow-up visit usually between 3 and 6 months postoperatively. Institutionalised patients were reviewed at CTO only if needed.

The radiographs, antero-posterior and lateral views, were evaluated by a single observer (AJB). Pre-operative radiographs were used to classify the fractures according to the AO/ASIF system [42]. The fractures classified as 31-A1 were considered stable [43,44].

Quality of fracture reduction was assessed on postoperative radiographs. For the reduction to be considered unsatisfactory there was misalignment on the antero-posterior radiograph of more than 10 mm of any fragment, 10° of varus/valgus angulation, and/or more than 20° of angulation on the lateral radiograph. Displacement of the lesser trochanter was disregarded.

Delayed union was defined as persistent pain and no sign of bridging callus after 4 months postoperatively. Non-union was defined as persistent pain and no sign of bridging callus 6 months postoperatively [45]. Avascular femoral head necrosis (AVN) was defined as pain over the groin on weight-bearing correlated with radiographic findings: subchondral fracture, segmental or total collapse of the femoral head accompanied by secondary osteoarthritic changes [46]. Revision as a cause for surgery has been defined as a secondary surgery with Gamma Nail following a failure of previous fixation in trochanteric fracture. Traffic accident and fall from height as fracture cause was defined as high-energy trauma.

The records for mean operating time, the intraoperative blood loss, and the level of surgeon experience were incomplete and not retrievable.

The following complications were recorded: general complications defined as medical and anaesthetic complications during surgery, technical complications during surgery, and postoperative complications defined as fracture-related problems after surgery.

The institutional review board at CTO gave ethical approval before the study was commenced. Due to the retrospective nature of the study no burden or risk was imposed on the patients.

### Statistical analysis

Results were tabulated and statistically analysed by using the SPSS (version 11.5, SPSS Inc. Chicago, Illinois, USA). The case report form and the corresponding variables were uniquely generated for our patient population and the questions adapted with help of patient notes samples before starting the study. Frequency and percent distributions were presented in tabular form for categorical variables. When data were missing, the remaining set of patients has always been validated by sex and age versus the total group of patients and when appropriate also for fracture pattern or fracture cause. Comparative analysis was performed by using the Chi-Square test for nominal and ordinal variables by evaluating frequencies within the groups with the method of cross tabulation. Before analysing continuous variables, the data sets were tested for normality by performing the Shapiro-Wilk test. When distribution was considered to be normal, for independent samples the Student's T test was performed; otherwise the Mann-Whitney test was used. Statistical significance for all tests was set at p-values less than 0.05. For normally distributed continuous variables, mean values, standard deviations (SD) and 95% confidence intervals (CI) of the mean are shown. For non-normal distributed continuous variables, median, range and interquartile ranges (IQR) are displayed.

## Results

### Demographics

In total, 3066 consecutive patients treated with Gamma Nails were identified. Of these 2255 were women (73.5%), ratio female: male 2.7. Median age was 81 years, ranging from 14 to 106 years, with an IQR of 16 years. In the overall patient population as well as in most of the subgroups, the variable "age" showed always a significantly skewed distribution (p < 0.001, Shapiro-Wilk Test).

The proportion of females and males did not change over the years. Left-sided fractures were present in 50.7% of the patients. For both genders, the number of fractures increased with age to peak between 81 and 90 years. In age groups 11-60 years males were predominant (ratio female/male 0.4). 53.0% of all male fractures occurred before the age of 70, but only 13.8% in women.

Three dominant fracture causes were identified: a simple fall (88.1%), road traffic accident (5.1%), fall from a height (3.0%) and other causes (3.8%). Traffic accidents were responsible for fractures in 44.0% of the patients up to the age of 40, after this age a simple fall was the most common cause for both genders. High-energy trauma accounted for 7.2% of all fractures and 1.5% of fractures were seen in multitrauma patients. Ten patients had open fractures.

**Figure 1:**
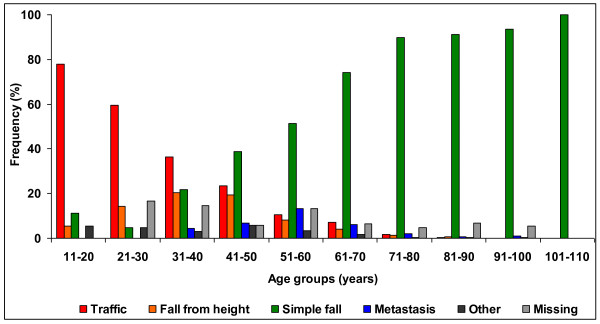
**Relative fracture cause distribution in age groups for both genders**.

**Figure 2:**
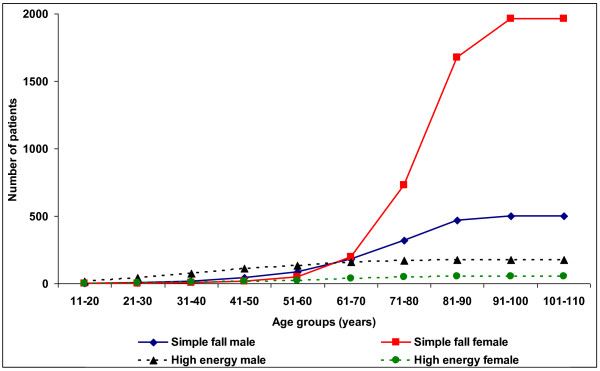
**Comparison of cumulative incidence in high-energy trauma vs. simple fall in age groups for both genders**.

The fracture aetiology did not change during the study period. Males in all age groups were more likely than females to sustain a fracture from high-energy trauma (traffic accident or fall from a height) 21.3% vs. 2.3% (p < 0.001). This is most obvious in ages under 21 where none of the female fractures were due to traffic accidents (Table 2). In absolute numbers, high-energy fractures were fairly constant within males up to age of 70 years, varying between 24 and 37 patients annually (Table 3). Low energy fractures were the most common fracture cause after the age of 50. In females the rate of high-energy fractures in all age groups was low and its contribution is obscured by the dramatic increase in fragility fractures after the age of 70 (Figures 1 and 2). Nevertheless, 46.0% of female high-energy fractures occurred in ages 61-80 years.

**Table 2:** Distribution of injury cause in age groups in women.

Age groups	Traffic	Fall from height	Simple fall	Revision	Metastasis	Other	Missing	Total	%
11-20	0	0	1	3	0	1	0	5	0.2
21-30	5	1	0	2	0	0	4	12	0.5
31-40	3	2	3	0	3	1	1	13	0.6
41-50	2	5	14	0	3	1	2	27	1.3
51-60	4	1	32	1	12	2	9	61	2.7
61-70	10	4	147	5	11	1	15	193	8.6
71-80	7	3	533	9	12	3	24	591	26.2
81-90	2	3	946	13	7	3	69	1043	46.2
91-100	0	0	287	2	3	1	15	308	13.6
101-110	0	0	2	0	0	0	0	2	0.1
Total	33	19	1965	35	51	13	139	2255	100
%	1.3	1.0	87.1	1.5	2.3	0.6	6.2	100	

**Table 3:** Distribution of injury cause in age groups in men.

Age groups	Traffic	Fall from height	Simple fall	Revision	Metastasis	Other	Missing	Total	%
11-20	14	1	1	0	0	0	0	16	2.0
21-30	20	5	2	8	0	2	3	40	5.0
31-40	22	12	12	3	0	1	9	59	7.3
41-50	22	15	26	6	4	5	4	82	10.1
51-60	11	11	42	4	7	3	10	88	10.8
61-70	14	10	98	4	9	5	6	146	18.0
71-80	5	6	139	5	5	0	12	172	21.2
81-90	1	4	148	3	1	1	13	171	21.1
91-100	0	0	33	0	0	0	3	36	4.4
101-110	0	0	1	0	0	0	0	1	0.1
Total	109	64	502	33	26	17	60	811	100
%	13.4	7.9	61.9	4.1	3.2	2.1	7.4	100	

Stable fractures (AO/ASIF 31-A1) constituted about one third of all fractures and grossly unstable fractures (AO/ASIF 31-A.3) constituted 12.1% (Table 1). Males were slightly overrepresented with unstable fractures. Patients under the age of 60 were more likely to sustain an unstable fracture than patients over 70 (62.5% vs. 59.6%, p < 0.005). The fracture distribution did not show any yearly variations.

On admission, 26.3% of the patients had no other medical condition, 34.2% had one co-existent disease, and 36.3% had 2-3 additional medical conditions, while 3.2% had more than 3 co-diseases. Almost half of the patients (48.0%) were socially independent. A prior contra-lateral hip implant was present in 10.2% of the patients.

### Treatment

The SGN was implanted in 1623 patients, the TGN in 933 patients and LGN in 473 patients. In 37 patients other nail types were used (Gamma-Ti Nail, Long Gamma-Ti Nail, Dyax Asiatic Nail). The LGN patients were significantly (p < 0.001) younger (median age 70 years, range 14 to 98 years, IQR 35 years) compared to patients in the SGN/TGN group (median age 82 years, range15 to 106 years, IQR 14 years). Furthermore, the LGN patients had, to a higher degree, sustained high-energy fractures (p < 0.001): traffic accidents (16.3%) and fall from height (9.4%). 20.1% of LGNs were used in pathological fractures and 8.6% for revision surgeries.

Fracture reduction was considered by the investigator to be satisfactory in 84.7% and unsatisfactory in 5.7% of cases, information for 9.6% was missing. In only three patients an open reduction was performed. According to the surgical notes, the surgeon found insertion of the nail to be difficult for 3.1% of the nails in the SGN group, 1.0% in TGN group and 5.3% in the LGN group. In 4.1% of all patients distal locking was not performed because of perceived good stability of the fracture or accidentally by missdrilling. Enhanced fixation (screws, cerclage wires, bone grafting) was used in 17 patients (0.6%). In 18 patients (0.6%) the fixation was believed to be insufficiently stable and full weight bearing was not allowed.

At discharge, 6.2% of the patients were sent home and required no additional assistance. Twenty percent were discharged to home with nursing or to a rehabilitation institution (37.1%) or a nursing home (22.6%). The rest (14.3%) were sent to other institutions as dictated by other medical conditions. One-hundred-and-forty-one patients (4.6%) died before hospital discharge.

### General complications

Altogether, 172 general complications (5.6%) were identified. Five patients had severe intraoperative complications related to anaesthesia; one of them deceased during surgery. One-hundred-and-sixty-seven complications (5.4%) were recorded postoperatively: 119 cardio-respiratory problems, 13 lung embolisms, 12 deep vein thromboses and 23 other medical complications (e.g. sepsis, renal insufficiency or stroke).

### Intraoperative fracture related complications

We observed 137 (4.5%) fracture related complications during operation. Difficulties with distal interlocking resulted in additional perforations of cortices or placement of the distal locking screw outside the nail in 104 patients (3.4%). Thirty-one of these occurred with free hand distal interlocking in LGNs. Targeted distal nail locking in short nails (SGN and TGN) resulted in 8.6% misdrillings until 1993. With the introduction of a new radiolucent targeting device in 1994, the rate of this complication dropped significantly to 1.1% (p < 0.001).

Intraoperative fractures were noticed in 17 cases (0.5%) (10 SGNs, 1 TGN and 6 LGNs). Thirteen intraoperative fractures occurred in the lateral cortex of the proximal femur (9 SGN, 1 TGN and 3 LGN). Another four patients sustained distal anterior cortical perforation by the tip of the nail (3 LGN, 1 SGN). In five of these 17 cases the surgeons noticed an aberrant shape of the femur due to rachitic deformity, Paget's disease or trisomy 21 syndrome.

Sixteen surgeon-related problems were recorded; mal-reduction of the fracture or poorly positioned implant in 12 patients that led to second surgical procedure to avoid later complications, guide wire perforation into the pelvis in two cases, lag screw perforation into the acetabulum in one case and one case of lost set screw in the soft tissue.

### Postoperative fracture related complications

Postoperatively and during follow-up 189 complications (6.2%) were detected (Table 4). We observed 19 postoperative femoral shaft fractures (0.6%). Thirteen of the 19 occurred with SGN, five with TGN and one with LGN. Fifteen were observed within less than three months postoperatively. One fracture was revised with a GK femoral nail and the others with LGN. Correlation between femoral shaft fractures and distal locking problems, age, fracture pattern or nail type could not be shown. There were nine cases of distal interlocking screw problems (screw breakage and screw backing out). In total, statistically significant fewer complications were seen in the TGN group compared with the SGN group. There were 105 patients (6.5%) in SGN and 32 patients (3.3%) in TGN group (p < 0.001).

**Table 4:** The analysis of fracture-related postoperative complications and their treatment.

Fracture related postoperative complication	Number of patients	Frequency (%)	Treatment	Number of patients
Cut out	57	1.85	THR	17
			Nail change	5
			Nail removal	8
			Lag screw removal	1
			No intervention	26
Infection	46	1.5	Nail removal	13
			Lavage	6
			THR	3
			No intervention	24
Femoral shaft fracture	19	0.6	Long Gamma Nail	19
Avascular head necrosis	17	0.5	THR	10
			Nail removal	1
			No intervention	6
Delayed healing/non-union	41	1.5	Nail dynamisation	32
			Bone grafting	10
			Exchange nailing	3
			THR	2
			Set screw removal	1
Distal locking screw problems	9	0.3	Screw removal	2
			No intervention	7

### Implant removal

Two-hundred-and-twenty-nine implant removal procedures were carried out. Eighty-seven nails were removed because of the complications described above. These patients were significantly (p < 0.001) younger (median age 73 years, range 30 to 93 years, IQR 22 years) than rest of the study group. Persistent pain warranted nail removal in 30 patients, in four cases distal locking screws were removed and in two cases the lag screw was exchanged. Additionally 106 nails (3.4%) were removed for reasons other than complications (patient desires, low age). The LGN was removed most frequently (11.8%), followed by SGN (2.3%) and TGN (1.3%). The nails were removed in 9.6% of males and only in 1.2% of females; this difference being strongly statistically significant (p < 0.001). Considering fracture type, Gamma Nails were removed in complex diaphyseal fractures (AO/ASIF 32-C) most frequently, 15 out of 25 implants (60%), followed by subtrochanteric and simple diaphyseal ones (AO/ASIF 32-A and B, 16/158, 10.1%), complex intertrochanteric (AO/ASIF 31-A3.3, 19/229, 8.3%) and simple intertrochanteric ones (AO/ASIF 31-A3.2, 6/78, 7.7%).

Without new trauma, eight fractures occurred in the trochanteric region within two weeks after nail removal in healed fractures. These patients were significantly (p < 0.001) younger (median age 58 years, range 20 to 77 years, IQR 32 years) than the rest of the study group. In three cases, the re-fracture followed the primary fracture line.

## Discussion

The most important finding of the present study is the low rate of fracture related complications with the use of the Gamma Nails in the treatment of trochanteric hip fractures compared with the literature [16,20,47-49].

In the present study, the rate of a subsequent fracture of the femoral shaft was extremely low (0.6%). Cut-out through the femoral head and AVN that led to revision were seen at a rate of 1.4%. General medical complications were seen in 5.6% of the patients, which is comparable with other studies [29,49].

Fracture of the femoral shaft is a serious complication that has been reported more frequently for the Gamma Nail than with other devices used for the treatment of trochanteric hip fractures. High incidence (6-17%) of a femoral shaft fracture after Gamma nailing was observed in earlier studies [6,14,50-52]. It was believed that this was due to specifics of the nail design of the first generation Standard Gamma Nail, such as the length, the valgus curvature and the distal diameter. It has also been suggested that there is a disparity between the design of the nail and the geometry of the bone resulting in the increased stiffness of the bone-implant system [14,53]. Inappropriate placement of the distal locking screws [6] and/or misdrilling could also be responsible for some of these complications. Incorrect reaming or excessive tightening of the distal interlocking screws [14,54] as well as the inappropriate handling of the implant such as the use of a hammer or insufficient reaming [3,53,54] could increase the risk for this complication. These are factors, which can all be controlled by proper training and more surgical experience [28]. In addition, intraoperative fractures may have passed unnoticed to present later as a femoral shaft fracture [29].

In accordance with other studies [38,48], most of the femoral shaft fractures occurred within three months postoperatively. However, the low rate of femoral shaft fracture in the present study (0.6%) is in contrast to other more recent reports on the SGN, e.g. 2.0% [16], 5.0% [48] or on other nail designs such as Proximal Femoral Nail (PFN, Synthes Switzerland); 2.0% [49], Proximal Femoral Nail Alpha (PFNA, Synthes Switzerland): 2.2% [55]. On the other hand in a study of 1000 patients [28] and in a study on the second-generation Gamma Nail (TGN) [5] this complication had a low rate of 1.1% and 0% respectively. A recent meta-analysis [56] clearly shows that newer design of the intramedullary devices and more surgical experience reduce the risk of the subsequent femoral shaft fracture.

We attribute the low rate of the postoperative femoral shaft fractures in this report to strict adherence to the original surgical technique at this study centre. Regardless of the implant, it appears that there is an increased rate of implant-related femoral shaft fractures after a hip fracture, particularly ipsilateral ones. The hip fracture population is fracture-prone and the consequences of a previous hip fracture such as diminished mental health score, altered hip- and femoral biomechanics, postoperative osteoporosis and increased likelihood of a new fall makes the patient susceptible to new injuries. It can therefore be assumed that there is a "baseline" level of postoperative femoral shaft fractures whatever method has been used to treat the hip fracture. This level appears to be in the range from 0% to 0.5% for nail fixation [5,49] and between 0.6% [48] and 1.2% for plate fixation [57]. Incidence above this level may be due to specifics of the study group such as age, gender and fracture type or related to the choice of implant or the skills with which it is used. Despite the significant decrease in distal locking problems after the introduction of a new targeting device in 1993 we could not show a further decrease in postoperative femoral shaft fractures indicating that a proper technique was followed from the beginning. The study population at the CTO appears to have reached the baseline level for this complication.

In the present study, the complication responsible for the highest number of revisions was cut-out through the femoral head (1.85%). The cut-out complication has been reported to be the most frequent mechanical mode of failure for internal fixation devices for treatment of trochanteric hip fractures [58-60]. The cut-out rate varies between 0 and 16% in the previous studies [16,28,32,49,61] and in older studies even up to 20% [62]. In previous reports, the revision incidence for these complications varies depending on the implant, with the Gamma Nail seeming to have a lower revision rate than plate fixation (Osnes *et al*. [48] plate fixation: 2.2%, SGN: 0.8%, Miedel *et al*. [29]: Medoff-SHS: 5.5%, SGN: 2.7%). We therefore suggest that more attention should be paid to this complication, which may be surgically preventable [16,63]. The incidence of cut-out in our study (1.85%) is comparable to the one reported by Kukla and co-workers (2.1%) [28].

Although the rate of femoral shaft fractures did not decline over the study period other improvements were seen. The distal locking problems decreased significantly (p < 0.001) after introduction of the second-generation targeting device at the end of 1993 (8.6% in early group vs. 1.1% in the late group). Significantly fewer fracture related complications were seen in the TGN group compared to the SGN group (p < 0.001). This probably reflects the newer design with increased anatomical conformity [14,53]. Apart from these improvements over time we could not find proof of a "learning curve" in contrast to other authors [28]. This may be explained by the CTO being the developing hospital for the use of the Gamma Nail, in particular the surgical technique, and has rigorously maintained the surgical principles and technique associated with the use of this implant.

Re-fracture of the trochanteric region was seen within two weeks after nail removal, for reasons other than revision, in eight patients (8/136 patients). The patients sustaining this complication were young (median age 58 years). Removal of the Gamma Nail should therefore be done with caution and the patient should be advised on partial weight bearing until consolidation [16,28,29].

Our data has allowed us to specify the cause for each fracture in a detail, which we believe, is unique. Gender and age specific patterns were clearly seen. Males are more likely than females to have a high-energy fracture. It is obvious that this is explained by different activity and labour pattern, not only osteoporosis. Nevertheless, 46.0% of female high-energy fractures occur in ages above 60, reflecting a high activity level in this age group. It is also striking that traffic accidents are the main cause for trochanteric fractures in the young up to the age of 40 years. The epidemiological data cannot be presented as incidence numbers, since we lack epidemiological background data for the catchment population. We have no reason to believe that it differs considerably from other countries in the region and data are in concordance with other reports [64,65].

This is by far the largest patient cohort treated with the Gamma Nails. Over 3000 consecutive patients at a single centre over a period of twelve years were analysed. The study period stretches from shortly after the market introduction of the implant until 2002. This also gave us the possibility of comparing two design generations of the implant. An additional strength of the study is that all radiological assessments, such as fracture classification, quality of fracture reduction and implant positioning were performed by a single observer in a limited time period (four months) limiting intra- and inter-observer bias, despite a possible risk for some systematic error.

Intra- and postoperative patient's notes were missing for 185 cases (6.0%). Only 1980 (64.6%) patients had at least one follow-up entry in our database. This important loss to follow-up can be explained by the retrospective character of the study and the long study period with varying methods in data collection. High mortality after trochanteric fractures and difficult surveillance of patients living at nursing homes contributed to the amount of missing data.

The quality of the radiographs was too variable to allow reliable assessment of osteoporosis grading according to Singh index [66], or tip - apex distance (TAD) measurement [58]. However, evaluation of position of the lag screw in the femoral head was possible for 2610 cases (85%).

The CTO had a defined patient catchment area increasing the likelihood for the return of the patients to the hospital, should a complication occur, thus enabling an accurate recording of postoperative events. Accordingly, it is unlikely that femoral fractures have been unreported particularly since these typically occur within three months after the primary operation [16,48].

## Conclusions

A number of patient-related factors predict the outcome after treatment of trochanteric hip fractures such as fracture geometry, age and gender of the patient [67] as well as dementia [68]. However, the surgeon is in control of fracture reduction, implant selection and implant placement, all of which must be optimized to ensure the success of the surgical intervention. We believe that this study supports the use of the Gamma Nail in trochanteric hip fractures leading to a low complication rate when the correct surgical technique is respected. As with any implant used for these fractures the main complication appears to be the cut-out of the lag screw through the femoral head, although at a low rate, while implant-related femoral shaft fracture is less of the problem. Further evaluation of hip fracture treatment should involve multi-centre randomised prospective trials comparing cephalo-medullary nails and sliding hip screws.

## Competing interests

CB, AS and AJ are employees of Stryker Osteosynthesis, Kiel, Germany, GT is and CE has been consultant to the same company. AJB was employed during the time of data collection by Stryker Osteosynthesis.

## Authors' contributions

AJB carried out the data collection, analysis and participated in manuscript writing. CB participated in study design performed statistical data analysis. AJ and CE participated in data analysis and manuscript writing. AS and GT participated in study design and coordination and help to draft the manuscript. All authors read and approved the final manuscript.

## Pre-publication history

The pre-publication history for this paper can be accessed here:

http://www.biomedcentral.com/1471-2474/11/133/prepub
